# Endocytic motif on HIV-1 Env regulates cleavage status and antibody neutralization of cell-free and cell-to-cell infection

**DOI:** 10.1128/jvi.01542-25

**Published:** 2026-05-28

**Authors:** Dania M. Figueroa Acosta, Tony R. Valencia, Hongru Li, Benjamin K. Chen

**Affiliations:** 1 Division of Infectious Diseases, Department of Medicine, Icahn School of Medicine at Mount Sinai 377569, New York, New York, USA; The Ohio State University, Columbus, Ohio, USA

**Keywords:** human immunodeficiency virus subtype 1 (HIV-1), cell-free infection, endocytic recycling, HIV-1 envelope (Env), conformational states, neutralization, cell-to-cell infection, bNAbs

## Abstract

Endocytic recycling regulates the abundance of human immunodeficiency virus type 1 (HIV-1) envelope glycoprotein (Env) on the cell surface, where Env comprises both cleaved and uncleaved forms. In contrast, released virions are enriched for cleaved Env. Because cell-free infection depends on virion-associated Env, and cell-to-cell infection is initiated by cell-surface Env, we examined whether Env endocytosis alters the balance of cleaved and uncleaved Env available for antibody recognition and thereby influences neutralization sensitivity in each transmission mode. To address this, we engineered an endocytic-deficient (ASPI-Env) and a cleavage-deficient (SEKS-Env) Env in a transmitted/founder clade B background. We compared them with wild-type Env using surface immunoprecipitation, ratiometric antibody binding, and neutralization assays. Disruption of the endocytic motif increased surface exposure of uncleaved Env and shifted the antigenic profile toward an uncleaved Env phenotype. Infection by cell-free virus particles carrying ASPI-Env showed increased sensitivity to antibodies that preferentially recognize uncleaved or open Env conformations (b12 and 17b), while its sensitivity to the cleaved Env-preferring epitope recognized by PGT151 was similar to wild type. In contrast, cell-to-cell infection showed minimal changes in neutralization sensitivity across the antibody panel, except for b12, which exhibited increased potency and greater inhibition of cell-to-cell HIV-1 transfer. Additionally, uncleaved SEKS-Env functioned in a CD4-dependent virion transfer assay, indicating a previously unappreciated ability to initiate cell-to-cell viral transfer. Together, these findings suggest that Env endocytosis shapes the heterogeneous pool of Env engaged in infection, thereby influencing antibody engagement, with important implications for HIV-1 vaccine strategies.

**IMPORTANCE:**

Env endocytosis shapes the antigenic landscape of HIV-1 by influencing the cleavage state of Env on both cells and virions. Env lacking a dominant internalization signal displays more uncleaved Env at the cell surface, thereby enhancing recognition by antibodies that preferentially target open or uncleaved conformations. Notably, cleavage-deficient Env can support CD4-dependent, cell-to-cell viral transfer, indicating that uncleaved cell surface Env can participate in early events of cell‑to‑cell infection. Together, these results suggest that Env sorting pathways influence cleavage-associated antigenicity and underscore the importance of considering both cleaved and uncleaved Env conformations in vaccine and cure strategies.

## INTRODUCTION

Human immunodeficiency virus spreads through both cell-free virions and direct cell-to-cell transmission across virological synapses formed when Env on infected donor cells engage CD4 on neighboring target cells (1–4). While Env mediates both pathways, cell-to-cell infection is notably more resistant to broadly neutralizing antibodies (bNAbs) than cell-free infection even at high antibody concentrations (3, 5–8). This reduced sensitivity has been proposed to reflect, at least in part, differences in the antigenic state of Env during the two modes of transmission (5, 9).

Env is a conformationally dynamic protein that spontaneously transitions among closed, intermediate, and open states (10). Cleavage can influence the state Env adopts by stabilizing the closed prefusion conformation, making cleaved Env less flexible than uncleaved Env, which more readily samples open states (11, 12). As a result, cleavage affects bNAb binding by altering epitope exposure (13–16). Additionally, cleavage limits access to glycan-modifying enzymes in the Golgi, shaping Env’s glycan composition (17). Because glycans account for roughly half of Env’s mass and many bNAbs recognize glycan-dependent epitopes (18–22), changes in glycan processing can further modulate antibody recognition. Importantly, virions are enriched for cleaved Env, whereas the cell surface displays a mixture of cleaved and uncleaved forms (23, 24). These differences create distinct antigenic landscapes, which may influence how antibodies engage Env during cell-free vs cell-to-cell transmission.

The selective enrichment of cleaved Env on virions is thought to result from selective trafficking processes during viral assembly, in which Env’s cytoplasmic tail (CT) plays a key role. Env’s CT contains a membrane-proximal tyrosine-based motif (YXXΦ) that mediates clathrin-dependent endocytosis of Env (25–27) and has been well studied as a key Env trafficking signal. Disruption of this motif alters Env internalization (8, 28–30) and neutralization sensitivity in an antibody-dependent manner (8), indicating a role in shaping antigenic presentation. Similarly, mutations in the endocytic motif of simian immunodeficiency virus (SIV) attenuated replication and delayed depletion of mucosal T cells, suggesting that changes in Env trafficking can influence immune recognition and pathogenesis (31, 32). In support of this, CT truncations have been shown to expose epitopes that are normally occluded in the native conformation (33), reinforcing the idea that CT integrity broadly affects Env antigenicity. Collectively, these findings indicate that endocytic trafficking of Env plays a critical role in shaping Env antigenicity; however, whether this process also regulates Env cleavage and glycan processing, and thereby contributes to antigenic differences between cell-free and cell-to-cell infection, remains unresolved.

Here, we hypothesized that Env endocytosis influences the antigenic state of cell-surface Env by modulating its cleavage, thereby altering antibody recognition during cell-free and cell-to-cell infection. To test this, we engineered an endocytic-deficient Env (ASPI-Env) and a cleavage-deficient Env (SEKS-Env) in a transmitted/founder (T/F) clade B HIV-1 background. Comparing ASPI-Env to SEKS-Env allowed us to determine whether changes observed in the endocytic mutant reflected a shift toward an uncleaved, open Env conformation. We evaluated these variants alongside wild-type Env using biochemical and neutralization assays to assess cleavage status and antibody sensitivity. Our findings reveal that Env endocytosis is a key determinant for maintaining cleavage-associated antigenicity and modulating epitope exposure, with direct implications for how Env heterogeneity shapes antibody recognition during HIV-1 infection.

## MATERIALS AND METHODS

### HIV-1 Env mutant clones

Transmitted/founder (T/F) HIV-1 QH0692 Env (ARP Cat. 1227, Drs. David Montefiori and Beatrice Hahn) was cloned into two pNL4-3-based fluorescent reporter backbones: the mCherry fluorescent NL-CI_NL4-3_ construct (34) and the Gag-iCherry construct, as previously described (35). To generate an endocytic-deficient mutant (ASPI-Env), a tyrosine-to-alanine substitution was introduced at residue 724 within the YXXΦ endocytic motif (YSPI to ASPI) using CloneAMP (Takarabio, Cat. 639298). A cleavage-deficient mutant (SEKS-Env) was created by mutating the primary furin cleavage site arginines to serines (REKR to SEKS) at positions 519 and 522. Mutant Env genes were inserted into the pNL4-3 molecular clone using EcoRI and MluI restriction sites with the In-Fusion HD Cloning Kit (Clontech Labs, Cat. 3P639648). For the Gag-iCherry backbone, the vector was digested with EcoRI-HF and XhoI-HF prior to insertion of mutant Env sequences by Gibson assembly (New England BioLabs, Cat. E5510S). All constructs were verified by Sanger sequencing across all amplified regions.

### Cell culture

293T cells were obtained from the American Type Culture Collection (ATCC). RevCEM-D4 (ARP-13437) and Jurkat E6 (ARP-177) cells were obtained from the NIH Reagent Program from Dr. Alex Sigal and Dr. Arthur Weiss, respectively. Primary CD4^+^ T cells were isolated from leukocyte packs obtained from the New York Blood Center and enriched from peripheral blood mononuclear cells using the CD4^+^ T cell Isolation Kit according to the manufacturer’s instructions (Miltenyi Biotec, Cat. 130096533). All cells were cultured as previously described in Durham et al. and Li et al. (8, 36). Primary cells were activated by supplementing media with 4 µg/mL of Phytohemagglutinin-L (SigmaAldrich, Cat. 11249738001) and 50 IU/mL of IL-2 (Miltenyi Biotec, Cat. 130097746).

### Virus production and immunoprecipitation of cell surface Env

293T cells were transfected using PolyJet *In Vitro* DNA Transfection Reagent (SignaGen Laboratories, Cat. SL1006885 × 1 mL). After 48 h, cells pellets were collected by centrifugation at 500 × *g*. Culture supernatants containing virus were centrifuged at 1,000 × *g* to remove debris and filtered through a 0.45 μM filter. Cell-surface Env immunoprecipitation was performed as previously described by Zou et al. (23). Briefly, intact cells were incubated with 10 µg/mL monoclonal antibodies PGT151, b12, 35O22, 17b, and 2G12 for 1 h at 4°C. Antibodies were obtained from the NIH HIV Reagent Program, Division of AIDS, NIAID, NIH: Anti-Human Immunodeficiency Virus (HIV)-1 gp41/gp120 Monoclonal Antibody (35O22), ARP-12586, contributed by Drs. Jinghe Huang and Mark Connors; monoclonal Anti-Human Immunodeficiency Virus Type 1 (HIV-1) gp120 (2G12), ARP-1476, contributed by Division of AIDS, NIAID; monoclonal Anti-Human Immunodeficiency Virus Type 1 (HIV-1) gp120 Protein, Clone 17b (produced *in vitro*), ARP-4091, contributed by Dr. James E. Robinson. Monoclonal antibody b12 and PGT151 were also produced by Yurogen Biosystems using their in-house human IgG Fc and kappa constant constructs (37, 38). After rigorous washing, cells were lysed in IP lysis buffer (Thermo Scientific, Cat. 87787) supplemented with Protease Inhibitor Cocktail (Thermo Scientific, Cat. 78415) according to the manufacturer’s instructions. Immune complexes were isolated using Pierce Protein G Magnetic Beads (Thermo Scientific, Cat. 88847). Virus-containing supernatants were stored for p24 quantification and concentrated by ultracentrifugation through 6% OptiPrep (Sigma-Aldrich, Cat. D1556). Concentrated virions were lysed under the same conditions as cell lysates.

### p24 enzyme linked immunosorbent assay

The p24 ELISA assay was performed as previously described (39) using the HIV-1 p24^CA^ Antigen Capture Assay Kit (Frederick National Laboratory). Samples and standards were incubated overnight at 4°C, followed by staining with primary rabbit anti-HIV-1 p24 antibody (1:200) and alkaline phosphatase-conjugated goat anti-rabbit IgG secondary antibody (1:8,000; Jackson Immuno Research Labs, Cat. 111055144). Luminescence was measured using a Cytation 3 instrument (BioTek) with Gen5 Microplate Reader and Image Software. Standard curves were generated by nonlinear regression using Prism (GraphPad Software).

### Western blotting

Samples were denatured in Invitrogen NuPAGE LDS Sample Buffer (Fisher Scientific, Cat. NP0007) with 50 mM DTT (Thermo Scientific, Cat. A39255) according to the manufacturer’s instructions. After electrophoresis, proteins were transferred to polyvinylidene difluoride (PVDF) membranes using the iBlot 2 Transfer Stacks (Invitrogen, Cat. IB24001) and the iBlot 2 Gel Transfer Device (Invitrogen). Membranes were blocked in 5% milk in 0.01% Tween 20 in Tris-buffered saline. gp120 mAb (ARP-1511, Susan Zolla-Pazner, AIDS Reagent Program) was diluted 1:2,300 to probe for gp120 Env subunit, mAb Chessie 8 was diluted 1:2,000, and p24 Gag antibody (ARP-6457, Michael H. Malim, AIDS Reagent Program) was diluted 1:1,000. Peroxidase AffiniPure Donkey Anti-Human IgG (H + L) (Jackson Immuno Research Labs, Cat. 709035149) was diluted 1:50,000 and Goat Anti-Mouse Affinity Purified IgG (H&L) (Rockland, Cat. 61013190500) was diluted 1:6,000 to be used as the secondary antibodies. Blots were developed with SuperSignal West Femto Maximum Sensitivity Substrate (Thermo Scientific, Cat. 34095), and images were captured using a SynGene Imaging System.

### Ratiometric and single antibody assay staining

Jurkat cells were nucleofected with NLCI_QH0692_ using the Cell Line Nucleofector Kit V (Lonza, Cat. VCA-1003) and incubated overnight at 37°C. Live cells were enriched with Ficoll-Paque (Cytivia, Cat. 17144003). Cells were stained with 5 µg/mL of a fluorescently conjugated antibody (i.e., PGT151, b12, or 2G12) for 45 min at 4°C. Antibodies were conjugated to a fluorophore using the Alexa Fluor 488 or 647 Antibody Labeling Kit (Molecular Probes, Cat. A20186 and A20181). After washing, cells were stained with Live/Dead Fixable Violet (Molecular Probes, Cat. L34964) for 20 min at 4°C, followed by a 20-min fixation with 2% paraformaldehyde (PFA) (Electron Microscopy Sciences, Cat. 15710S) at 4°C. Samples were analyzed on an Attune NxT Flow Cytometer (Thermo Fisher Scientific). Event-level fluorescence intensities were exported from FCS Express Flow Cytometry Software for mCherry-positive cells. Antibody binding ratios were calculated as:


$$\left(\frac{PGT151\;FI\;for\;mCherry\;positive\;cells}{2G12\;FI\;for\;mCherry\;positive\;cells}\right)\;or\;\left(\frac{b12\;FI\;for\;mCherry\;positive\;cells}{2G12\;FI\;for\;mCherry\;positive\;cells}\right)$$

Outliers were removed using the interquartile range (IQR) method in R, and statistical analysis was performed in Prism.

### Single-round cell-free infection assay and neutralization

Virions were harvested and quantified as described above. Cell-free infection and neutralization assays were adapted from previous publication (8). Briefly, virus corresponding to up to 58 ng of p24 was incubated with 1 × 10^5^ RevCEM for 18 h. Cultures were then washed and replaced with complete RPMI media with 10 μM azidothymidine (AZT) (NIH HIV Reagent Program, NIAID, HRP-3485) to restrict infection to a single replication cycle. After 40 h, cells were stained with Live/Dead Fixable Violet or Green Dead Cell Stain Kit (Molecular Probes, Cat. L34964 and L23101), fixed in 2% PFA, and analyzed by flow cytometry. For neutralization studies, viruses were pre-incubated with monoclonal antibodies (mAbs) for 30 min at 37°C prior to addition to cells. As a negative control, target cells were pre-incubated with the anti-CD4 antibody Leu3a (BC Cell Analysis, Cat. 3P340853).

### Cell-to-cell infection assay and neutralization

The cell-to-cell infection assay was adapted from a previous publication (36). Jurkat T cells nucleofected with the NLCI_QH0692_ constructs served as donor cells, and RevCEM cells were used as target cells. Donor and target cells were dye-labeled with cell proliferation dye eFluor 670 and eFluor 450, respectively (Invitrogen Cat. 65084090 and 65084290). A total of 1 × 10^5^ donor cells were co-cultured with equal amounts of target cells for 18 h. Cultures were then washed and replaced with complete RPMI media containing 10 μM AZT. After 40 h, cells were stained with a live/dead cell stain, fixed in 2% PFA for 20 min, and analyzed by flow cytometry. Infection of target cells was quantified as mCherry-positive events within the eFluor 450-labeled population. For neutralization assays, donor cells were pre-incubated with Env-specific mAbs, and target cells were pre-incubated with Leu3a for 30 min at 37°C prior to co-culture.

### Cell-to-cell transfer assay and transfer-neutralization

Cell-to-cell transfer assays were performed as previously described (36) using Jurkat donor cells expressing HIV-1 Gag-iCherry constructs and RevCEM target cells. Donor and target cells were prepared as described for the cell-to-cell infection assay. Co-culturing was permitted for 6 h; a duration chosen to restrict the assay to CD4-dependent particle transfer rather than productive infection. Following co-culture, cells were washed and briefly treated with trypsin to remove surface-attached virions; trypsin was neutralized by adding complete RPMI media. Internalized viral particles were detected as mCherry-positive events within the eFluor450-labeled target-cell population. For transfer-neutralization experiments, donor cells were pre-incubated with Env-specific mAbs, and target cells were pre-incubated with Leu3a for 30 min at 37°C before co-culturing. Samples were analyzed by flow cytometry.

## RESULTS

### Comparison of endocytic- and cleavage-deficient Env mutants

**Fig 1:**
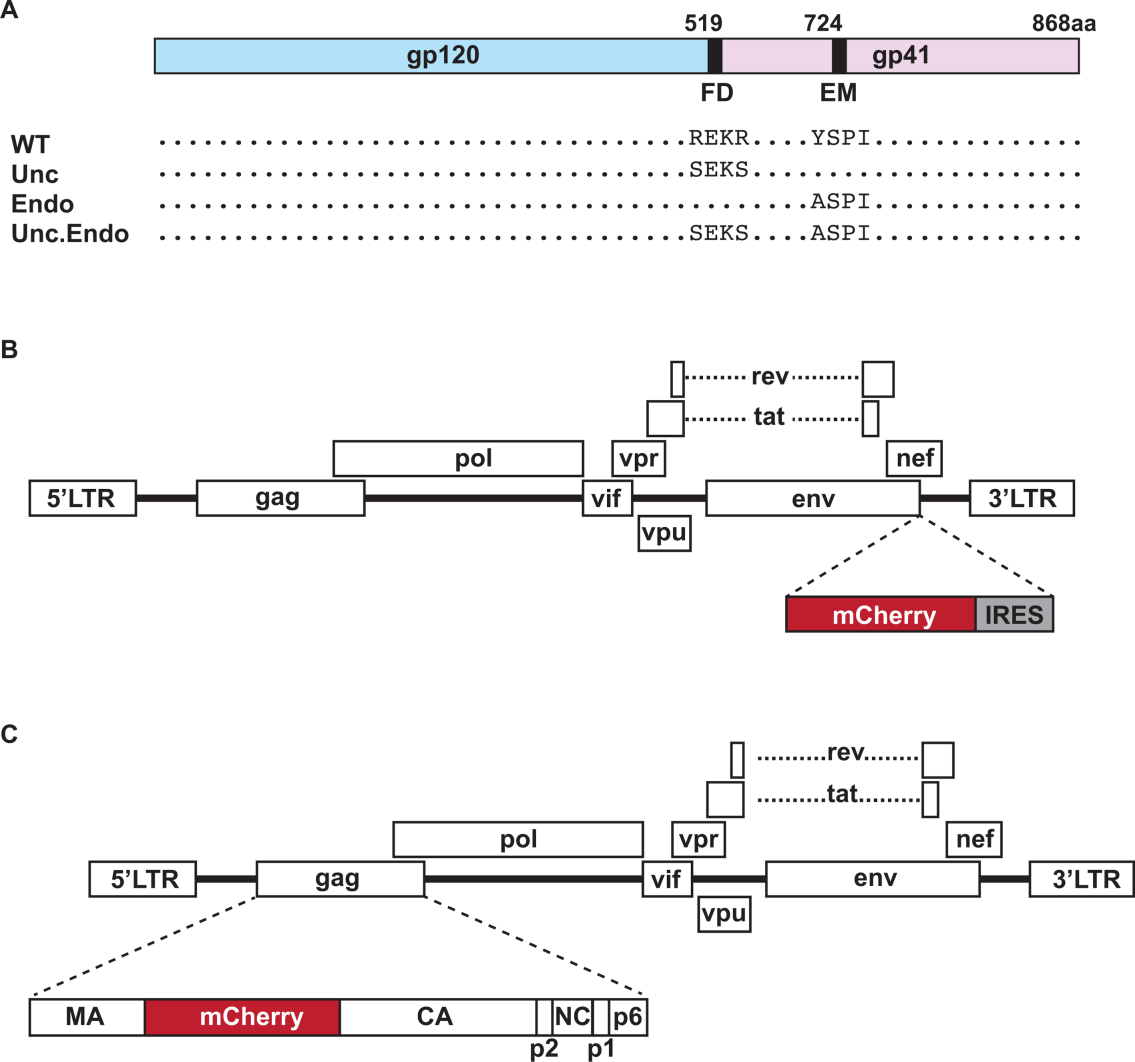
Endocytosis and furin cleavage site mutations within fluorescent reporter HIV-1. (**A**) Schematic representation of Env mutations in the furin cleavage site (FS) and main endocytic motif (EM). Proviral DNA of T/F QH0692 ENV was cloned into the NL43 backbone, producing an uncleavable (Unc), endocytosis-deficient (Endo), and double mutant (Unc.Endo) variants. (**B**) NL-CI construct includes an mCherry and internal ribosome entry site (IRES) upstream of the nef gene. (**C**) Gag-iCherry construct consists of an mCherry signal between the matrix (MA) and capsid (CA) genes.

To investigate whether Env endocytosis influences its cleavage state, we generated two HIV-1 Env mutants in the T/F HIV-1 QH0692 background: an endocytic-deficient Env (ASPI-Env) created by substituting the tyrosine YXXΦ motif with alanine (Y724A) and a cleavage-deficient Env (SEKS-Env), produced by mutating the furin cleavage site at the gp120-gp41 junction, ^519^REKR^522^, to SEKS (Fig. 1A). SEKS-Env served as a reference for uncleaved Env, enabling us to determine whether changes observed in ASPI-Env reflect a shift toward an Env population more representative of uncleaved, open conformations. These mutations were introduced into the fluorescent HIV-1 reporter NLCI (Fig. 1B) (34) and Gag-iCherry (35) (Fig. 1C) to enable parallel analysis of cell-free, cell-associated infection, and cell-to-cell viral transfer.

### Analysis of isoforms and glycosylation in Env mutants in cells and virions

**Fig 2:**
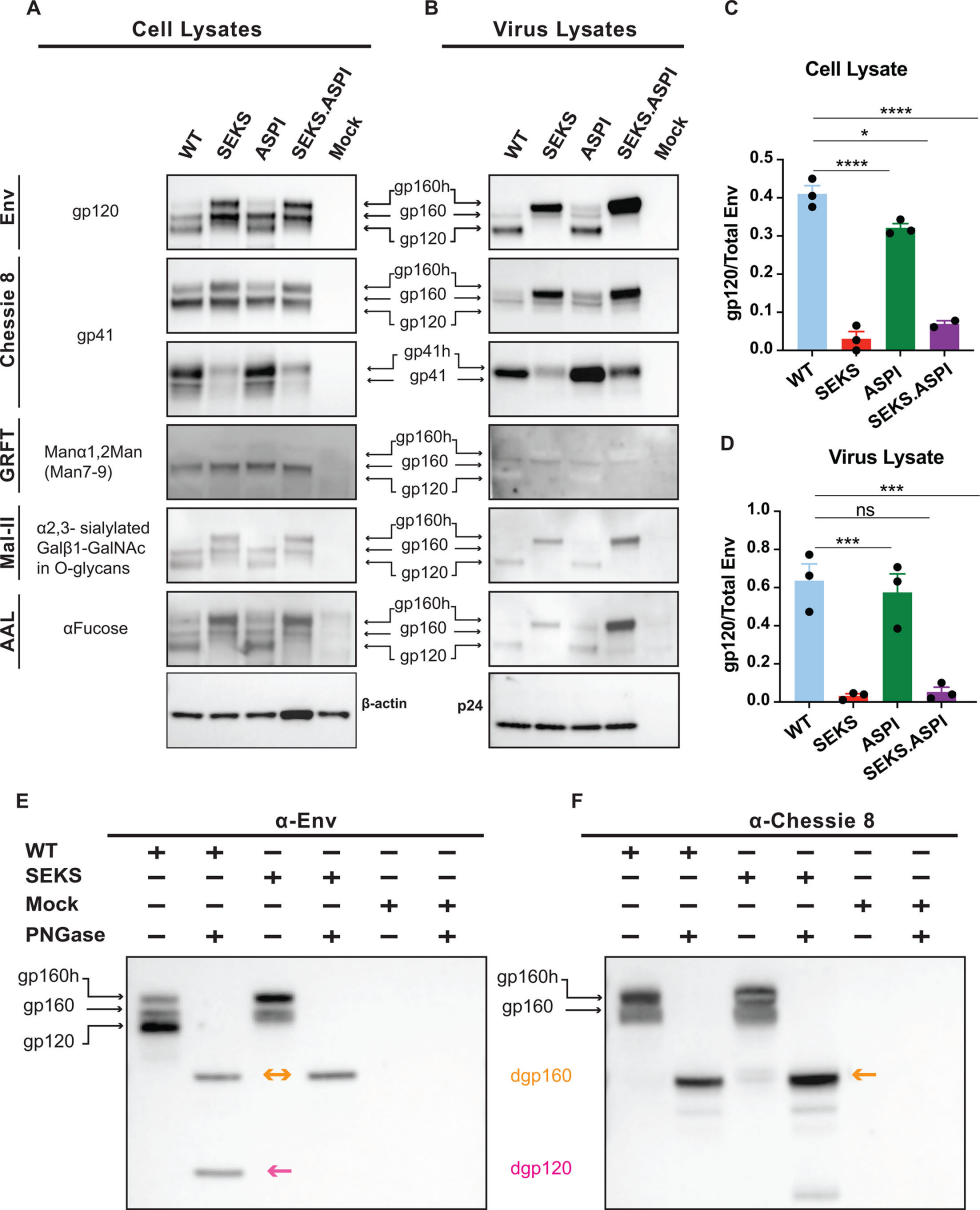
Expression of cleaved and uncleaved glycoforms of Env within the cell and on virions, and the impact of ASPI mutation in comparison to the uncleavable SEKS mutant. WT and mutant NLCI_QH0692_ were expressed in (**A**) 293T cells and (**B**) concentrated viral particles. Cell lysates were obtained from mock immunoprecipitation samples. Densitometric analysis of cleaved Env relative to total Env bands yields cleavage ratios for Env in (**C**) cell and (**D**) viral lysates. Densitometry data from three independent experiments, with standard error of the mean (SEM), are shown. Cell-lysate samples from WT and SEKS-Env expressing cells were treated with PNGase F to remove N-linked glycans and probed with (**E**) anti-Env and (**F**) Chessie mAb. Statistical significance was determined using Dunnett’s multiple comparisons test: *P* < 0.05 = *, *P* < 0.01 = **, *P* < 0.001 = ***, and *P* < 0.0001 = ****, ns = not significant.

We first examined how mutations in the endocytic motif and furin cleavage site affect total Env cleavage and glycosylation. Wild-type and mutant NLCI_QH0692_ constructs were expressed in 293T cells, and cell and viral lysates were analyzed by western blot to assess Env isoform abundance and glycan composition. Western blotting revealed distinct banding patterns among the mutants of T/F Env QH0692. WT- and ASPI-Env displayed three bands, whereas cleavage-deficient SEKS-Env and SEKS.ASPI-Env mutants showed only two (Fig. 2A). Probing with gp41 epitope monoclonal antibody, Chessie 8, confirmed that the upper bands corresponded to uncleaved Env (gp160), while the lower band represented cleaved Env (gp120) (Fig. 2A). We designated the upper gp160 band as gp160h, representing a higher-molecular-weight uncleaved Env, and the lower band as gp160. Notably, Chessie 8 also detected faint gp41 bands in the SEKS mutants (Fig. 2A), which may reflect residual Env cleavage or degradation products in cells or viruses. Regardless of origin, the absence of a clear gp120 band indicates that cleavage is defective in the SEKS mutants. In contrast, ASPI-Env showed a modest but statistically significant reduction in cleaved Env compared to WT-Env in cell lysates (Fig. 2A and C). Importantly, this loss was not observed in viral lysates, where gp120 incorporation remained comparable between WT- and ASPI-Env (Fig. 2B and D).

To determine whether the two gp160 bands are glycovariants, we blotted with lectins specific for high-mannose (Griffithsin, GRFT) (40, 41), sialylated (*Maackia amurensis* lectin II, MAL-II) (42), and fucosylated (*Aleuria aurantia* lectin, AAL) (43) glycans. GRFT bound selectively to the lower gp160 band, while MAL-II and AAL bound all isoforms, suggesting differences in glycan processing (Fig. 2A and B). Notably, AAL displayed stronger binding to the upper gp160 band despite Env staining showing that both gp160 isoforms were present at similar levels in SEKS-Env cell lysates (Fig. 2A). This suggests that the two species differ in glycan composition. To further confirm that these bands represent glycovariants, we treated cell lysates with PNGase F, which removes all N-linked glycans. After treatment, WT-Env samples collapsed from three bands to two, and SEKS-Env samples collapsed from two bands to one (Fig. 2E). The collapsed pattern in the SEKS-Env sample, which lacks gp120, suggests that both original bands were gp160 glycovariants. To confirm the identity of the bands in the WT-Env samples after treatment, we probed with gp41 reactive monoclonal antibody, Chessie 8. The strongest signal corresponded to the upper band, which we termed deglycosylated gp160 (dgp160), further confirming that the collapsed species in the SEKS-Env sample is gp160 (Fig. 2F). We, therefore, concluded that the lower band of the WT-Env treated sample was deglycosylated gp120 (dgp120). Together with the lectin binding, these results confirm that the heterogeneity between the two gp160 species reflects differential glycosylation. Interestingly, although both gp160 forms are present in SEKS-Env cell lysates at similar levels, gp160h is preferentially incorporated into virions (Fig. 2A and B). This suggests a mechanism exists whereby a particular glycosylation state correlates with Env packaging.

### Endocytic motif reduces cell surface expression of cleaved Env

To determine how the endocytic motif affects Env cleavage at the cell surface, we performed a surface immunoprecipitation assay using a focused panel of antibodies with defined dependencies on Env cleavage and glycan composition, summarized in Table 1. For clarity, we refer to these collectively as cleavage- or glycan-sensitive antibodies. PGT151 recognizes a quaternary epitope spanning the gp120-gp41 interface and binds only cleaved Env (38, 44), making it a direct reporter for surface-exposed gp120. In contrast, 35O22 also targets the gp120-gp41 interface but is not cleavage-dependent; its binding is enhanced when glycan maturation is impaired, and high-mannose glycans accumulate (45, 46). To assess epitopes that are more accessible on uncleaved Env, we included b12, a CD4-binding site antibody that has increased affinity for uncleaved Env (14, 15, 44, 47). Finally, 2G12, which binds an oligomannose epitope, was used to probe the connection between glycan composition and Env processing (14, 15, 48, 49).

**TABLE 1:** Epitopes of bNAbs that target cleavage- and glycan-sensitive epitopes^*a*^

Antibody	Epitope target	Cleavage preference		References
		Cleaved	Uncleaved	
PGT151	gp120-gp41 interface	+	−	(38, 44)
35O22	gp120-gp41 interface	+	+	(44, 46)
b12	CD4 binding site	+	++	(14, 15, 44, 47)
17b	CD4 induced	−	+	(50–52)
2G12	V3 glycan	Glycan-specific (high-mannose)		(14, 15, 48, 49)

^*a*^ − denotes no or minimal binding; + denotes preferential binding to cleaved or uncleaved Env, ++ denotes strong preferential binding to cleaved or uncleaved Env.

**Fig 3:**
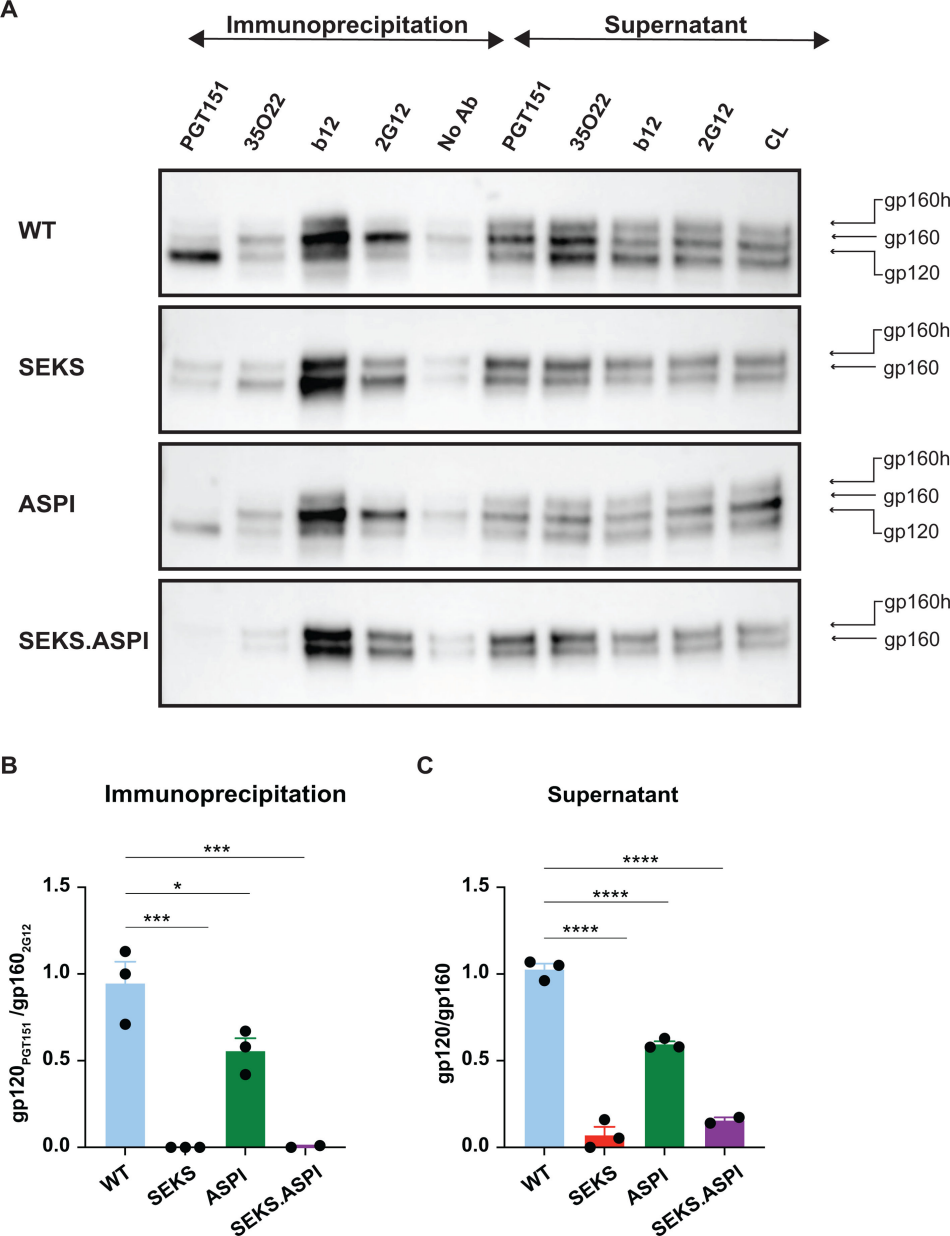
Immunoprecipitation of cell-surface Env with Abs that selectively recognize distinct isoforms of Env. (**A**) Cell surface Env was immunoprecipitated from 293T cells transfected with WT and mutant NLCI_QH0692_. Immunoprecipitation was performed with mAbs (PGT151, 35O22, b12, and 2G12). Western blotting was performed with anti-Env antibody gp120 (268-D IV). (**B**) Densitometry analysis for gp120 band (by PGT151) and gp160 band (by 2G12) provides a cleavage ratio of cell-surface Env. (**C**) Total Env cleavage ratio is calculated by performing densitometry analysis on gp120 and gp160 band in the cell lysates (CL). Densitometry data from three independent experiments with SEM are shown. Statistical significance was determined using Dunnett’s multiple comparisons test: *P* < 0.05 = *, *P* < 0.01 = **, *P* < 0.001 = ***, and *P* < 0.0001 = ****, ns = not significant.

Using these antibodies, we first examined the cleavage composition of surface Env in WT-Env-expressing cells. The IP revealed a heterogeneous mixture of cleaved and uncleaved Env at the cell surface, providing a baseline against which the endocytic and cleavage mutants could be compared. This was most evident with 35O22 which recovered both gp160 and gp120, consistent with its ability to recognize the gp120-gp41 interface independent of cleavage (Fig. 3A). As expected, PGT151 selectively pulled down gp120 (Fig. 3A), whereas b12 and 2G12 predominantly captured gp160 (Fig. 3A), reflecting their preference for epitopes more exposed on uncleaved or high-mannose rich Env. In ASPI-Env-expressing cells, PGT151 recovered markedly less gp120 than in WT-Env, indicating a reduction in surface-exposed cleaved Env when the endocytic motif is disrupted. To quantify this loss, we calculated the ratio of PGT151-bound gp120 to 2G12-bound gp160, which revealed a significant decrease in surface cleavage for ASPI-Env relative to WT-Env (Fig. 3B). This reduction paralleled the cleavage defect observed in cell lysates (Fig. 3C).

As expected, the SEKS mutation reduced PGT151 pulldown, further confirming the reduction of cleaved Env at the cell surface. Notably, however, IP with 35O22, b12, and 2G12 recovered both gp160 and gp160h from SEKS- and SEKS.ASPI-Env-expressing cells. This demonstrates that both uncleaved Env glycoforms can reach the plasma membrane, indicating that the preferential incorporation of gp160h onto virions observed in Fig. 2B is not the result of selective trafficking of gp160h to the cell surface. Instead, only a subset of surface Env is selected for virion assembly, suggesting that both the glycosylation and cleavage state of Env may contribute to Env sorting to assembly sites. This distinction reinforces the concept that the cell-surface Env pool is more heterogeneous than the Env population on virions and that endocytosis helps shape this surface composition.

### Endocytic mutant displays cleavage-associated antigenicity on the cell surface

**Fig 4:**
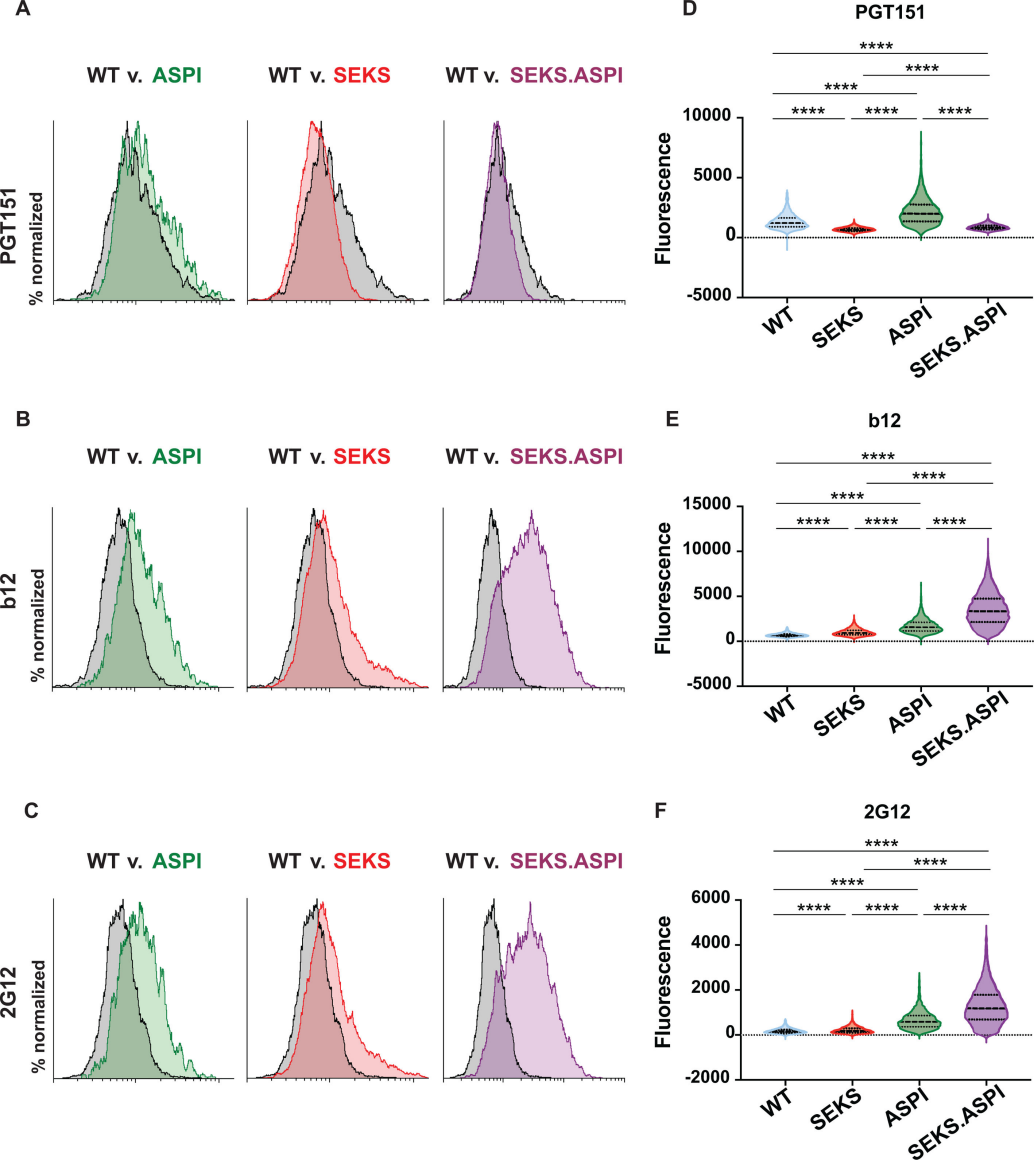
Single Ab staining of cell surface Env with cleavage- and glycan-sensitive Abs. Representative flow cytometry histograms show Ab staining of cell-surface WT and mutant NLCI_QH0692_ in Jurkat T cells with (**A**) PGT151, (**B**) b12, and (**C**) 2G12 mAbs. Corresponding quantification of fluorescence intensity for the HIV-positive cell population is shown as violin plots for (**D**) PGT151, (**E**) b12, and (**F**) 2G12. Event-level statistics were conducted across three independent experiments. Statistical significance was determined using Dunn’s multiple comparisons test: *P* < 0.05 = *, *P* < 0.01 = **, *P* < 0.001 = ***, and *P* < 0.0001 = ****, ns = not significant.

To determine how Env endocytosis affects the cleavage state of Env on T cells, we analyzed surface antibody binding to WT and mutant Env. PGT151 single staining shows extensive overlap with only a subtle rightward shift for ASPI-Env compared to WT-Env (Fig. 4A), consistent with the known increase in surface Env abundance upon disruption of the endocytic motif. However, when fluorescence was quantified across experiments, ASPI-Env displayed a significant increase in PGT151 signal (Fig. 4D). Because both measurements represent single-antibody staining, the increased PGT151 signal cannot distinguish between increased Env surface abundance, as has been previously observed for ASPI-Env mutants (8, 28–30), and changes in the proportion of cleaved vs uncleaved Env. In contrast, PGT151 staining of SEKS- and SEKS.ASPI-Env-expressing cells showed a clear and consistent loss of signal, reflected by a downward shift in both the histograms and fluorescence quantification (Fig. 4A and D). This decrease is expected as the SEKS mutation disrupts furin cleavage and eliminates gp120 expression on the cell surface. Staining with b12 and 2G12 revealed a more consistent pattern as all mutant Envs demonstrated increased antibody binding compared to WT-Env, reflected by a rightward shift in both the histogram and fluorescence quantification (Fig. 4B, C, E and F). Because b12 can bind both cleaved and uncleaved Env and has increased affinity for uncleaved Env, its enhanced staining in the ASPI-Env mutant (Fig. 4B and E) likely reflects a combination of increased total Env on the surface and greater exposure of epitopes characteristic of uncleaved Env. Similarly, because 2G12 recognizes the high-mannose glycan patch found on both cleaved and uncleaved Env, its increased binding (Fig. 4C and F) cannot distinguish between changes in the proportion of cleaved Env and increased surface Env abundance.

**Fig 5:**
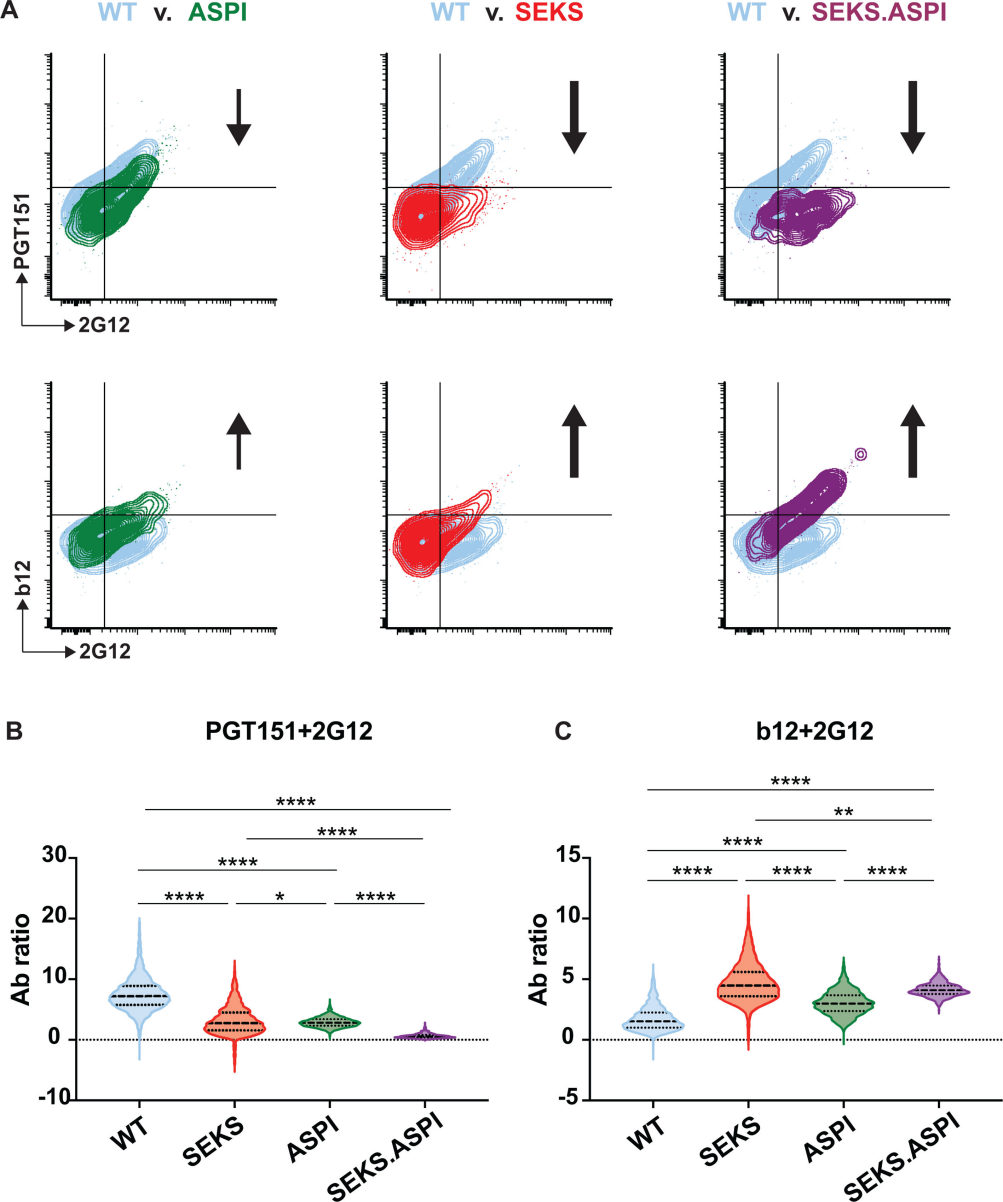
Dual fluorescence flow cytometry assay with cleavage- and glycan-sensitive Abs. (**A**) Representative flow cytometry dot plots show Ab staining of cell surface WT and mutant NLCI_QH0692_ in Jurkat T cells. Arrows represent the Ab binding shift. (**B and C**) The antibody binding ratio was calculated as the fluorescence intensity for each Ab pair in the HIV-positive population of cells. Event-level statistics were conducted across four independent experiments, as shown. Statistical significance was determined using Dunn’s multiple comparisons test: *P* < 0.05 = *, *P* < 0.01 = **, *P* < 0.001 = ***, and *P* < 0.0001 = ****, ns = not significant.

To overcome the limitations of single-antibody staining, in which changes in surface abundance can mask conformational differences, we performed a ratiometric antibody-binding assay using two-color flow cytometry. This approach measures PGT151 or b12 binding relative to that of the glycan-targeting 2G12 antibody, which has previously been used to normalize Env expression (13, 14, 53, 54). Antibodies were added simultaneously, as sequential staining yielded comparable binding patterns (data not shown). Relative to WT-, ASPI-Env-expressing cells demonstrated reduced PGT151 binding normalized to 2G12 binding (Fig. 5A), with an even more pronounced decrease observed for SEKS- and SEKS.ASPI-Env-expressing cells (Fig. 5A). As PGT151 recognizes a cleavage-dependent epitope, this reduction suggests that disrupting Env internalization diminishes the proportion of cleaved Env exposed on the cell surface. In contrast, ASPI-Env displayed increased b12 binding compared to WT-Env, mirroring the pattern observed for SEKS- and SEKS.ASPI-Env-expressing cells (Fig. 5A). Notably, although the proportion of b12-positive WT-Env-expressing cells appears low in the ratiometric assay, it is consistent with single-antibody staining. Quantification of the ratiometric binding assay confirmed that all mutant Envs exhibited significant loss of PGT151 binding (Fig. 5B) and corresponding increase in b12 binding relative to WT-Env (Fig. 5C). Together, these data demonstrate that disruption of the endocytic motif shifts the antigenic profile of cell-surface Env toward epitopes preferentially exposed on uncleaved Env and that this shift is unlikely to be explained solely by increased Env surface expression.

### Disruption of the endocytic motif increases accessibility of epitopes exposed on uncleaved Env in cell-free virions

**Fig 6:**
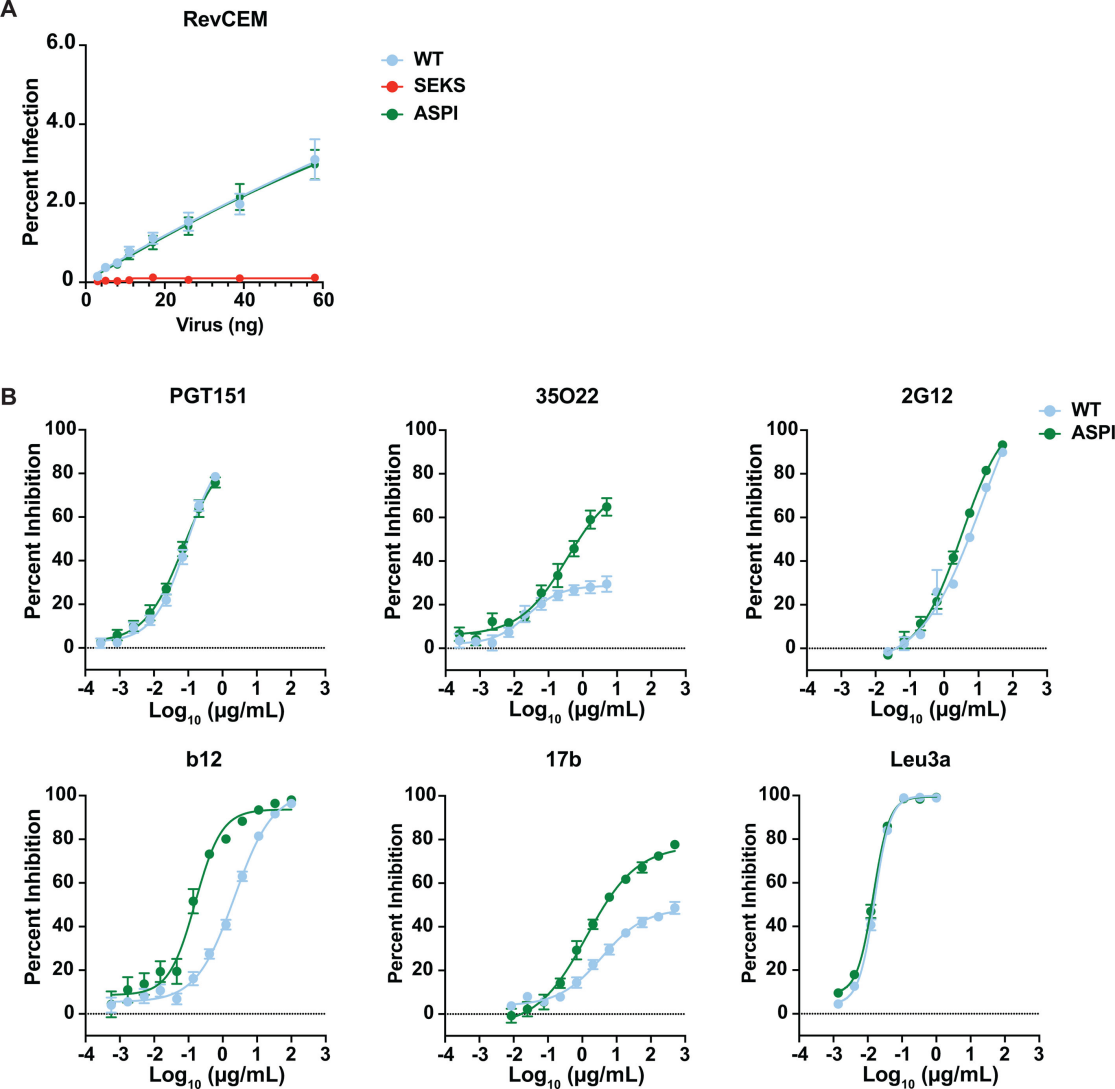
Neutralization assays of cell-free infection comparing WT vs endocytic motif mutant using cleavage- and glycan-sensitive Abs. (**A**) WT and mutant NLCI_QH0692_ cell-free virions produced in 293T cells were titrated onto RevCEM cells. p24 ELISA was used to determine virus concentration. (**B**) Neutralization of cell-free infection against WT and ASPI NLCI_QH0692_ by PGT151, 35O22, 2G12, b12, 17b, and Leu3a. The virus was incubated with mAbs for 30 min at 37°C before culturing with cells. The mean and SEM data are shown for at least three independent experiments.

We next evaluated whether impaired Env endocytosis alters the antigenic profile of HIV-1 virions by generating cell-free wild-type and mutant viruses (NLCI_WT_, NLCI_SEKS_, and NLCI_ASPI_). As expected, NLCI_ASPI_ virions remained infectious, consistent with prior reports (Fig. 6A) (55–57), whereas NLCI_SEKS_ virions were noninfectious (Fig. 6A) due to the absence of Env cleavage (58, 59). Next, we assessed neutralization sensitivity using our panel of cleavage- and glycan-sensitive antibodies. We also tested 17b, which recognizes a CD4-induced epitope characteristic of an open Env conformation, a state enriched on uncleaved Env (Table 1) (50–52). As a control, we included the CD4-binding antibody Leu3a, which inhibits infection by competing with Env for CD4 engagement. Leu3a showed no difference in neutralization between NLCI_WT_ and NLCI_ASPI_, indicating that CD4-dependent entry remained comparable between the two viruses (Fig. 6B). Neutralization by PGT151 and 2G12 was also similar for both viruses (Fig. 6B). In comparison, NLCI_ASPI_ showed increased sensitivity to neutralization by 35O22, b12, and 17b (Fig. 6B), consistent with enhanced exposure of epitopes associated with uncleaved, open Env. Reflecting this effect, 35O22 neutralized ~60% of NLCI_ASPI_ at 10 μg/mL compared to ~20% of NLCI_WT_ (Fig. 6B). Similarly, 17b neutralized ~80% of NLCI_ASPI_ at 1000 μg/mL, whereas only ~40% of NLCI_WT_ was neutralized (Fig. 6B). b12 neutralized 100% of both; however, its potency against NLCI_ASPI_ was increased, with a half-maximal inhibitory concentration approximately 10-fold lower than that of NLCI_WT_ (Fig. 6B). Together, these results suggest that even small increases in uncleaved Env incorporation can substantially shift the neutralization profile of cell-free HIV-1.

### Endocytic-deficient Env exhibits increased sensitivity to b12 during cell-to-cell infection

**Fig 7:**
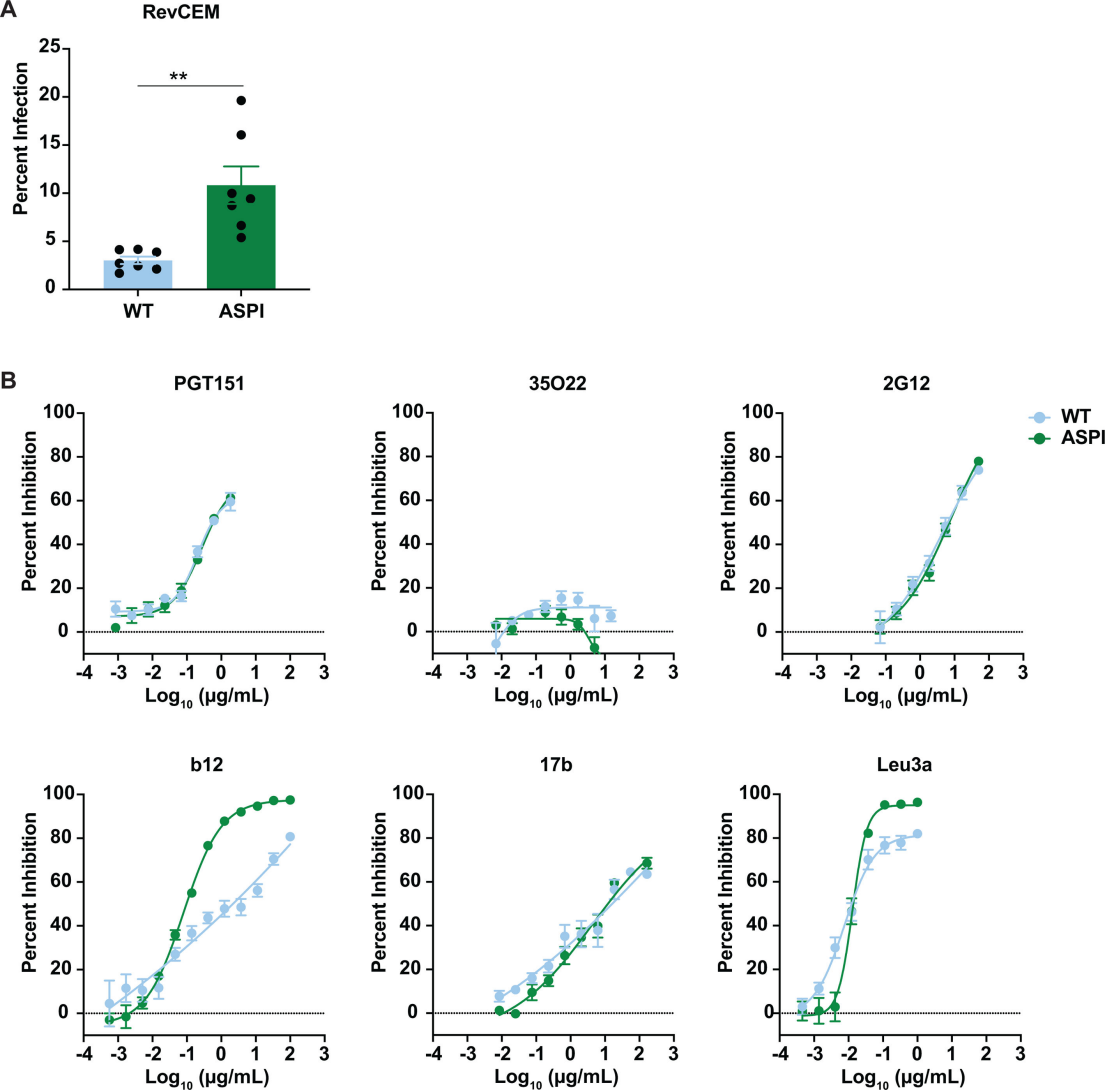
Neutralization assays of cell-to-cell infection with cleavage- and glycan-sensitive Abs. (**A**) Nucleofected Jurkat T cells expressing WT and ASPI NLCI_QH0692_ were cocultured with RevCEM cells. Nucleofection efficiency was determined by measuring mCherry expression in Jurkat T cells 24 h after nucleofection using flow cytometry. Nucleofection efficiency was normalized by spiking in mock nucleofected Jurkat T cells prior to co-culturing. (**B**) Neutralization of WT and ASPI NLCI_QH0692_ cell-to-cell infection by PGT151, 35O22, 2G12, b12, 17b, and Leu3a. The donor cells were incubated with anti-Env mAbs and target cells with Leu3a for 30 min at 37°C before co-culturing cells. All experiments shown represent at least three independent experiments. The mean and SEM data are shown for at least three independent experiments. Statistical significance was performed with an unpaired *t* test with Welch’s correction: *P* < 0.05 = *, *P* < 0.01 = **, *P* < 0.001 = ***, and *P* < 0.0001 = ****, ns = not significant.

Next, we examined whether the altered membrane cleavage ratio produced by the endocytic motif mutation influences antibody susceptibility to cell-to-cell infection. We used a donor-target coculture assay in which infection is driven by plasma membrane-embedded Env engaging CD4 to form a virological synapse (36). This assay has previously been shown to rely minimally on cell-free virus, as target cells exposed only to supernatants from infected cultures or separated by a transwell barrier are inefficiently infected (3, 4, 60). To ensure that differences in cell-to-cell infectivity reflected the effects of the endocytic motif mutation rather than variations in donor-cell expression levels, the donor population was normalized based on reporter (mCherry) expression. Both WT-Env and ASPI-Env donor cells mediated productive cell-to-cell infection (Fig. 7A). SEKS- and SEKS.ASPI-Env donors were not tested, as their lack of fusogenic activity prevents the productive infection required for readout in this assay.

Next, we tested the sensitivity of the cleavage- and glycan-sensitive antibodies discussed in the previous section to inhibit WT- and ASPI-Env-mediated cell-to-cell infection. ASPI-Env showed similar sensitivity to PGT151, 35O22, 2G12, and 17b compared to WT-Env (Fig. 7B). Notably, 35O22 did not inhibit cell-to-cell infection mediated by either Env (Fig. 7B) despite modest inhibition of WT cell-free virus (~20%, Fig. 6B). This observation is consistent with prior reports that cell-to-cell infection is generally more resistant to neutralizing antibodies (3, 5–8). In contrast, ASPI-Env was markedly more sensitive to b12 (Fig. 7B). Against WT-Env b12 exhibited a broad, non-sigmoidal inhibition curve, whereas inhibition of ASPI-Env revealed a sigmoidal pattern (Fig. 7B). This shift is reflected in the concentration of antibody required to achieve 80% inhibition: ~143 µg/mL was required for WT-Env, whereas ASPI-Env was neutralized at only ~0.5 µg/mL (Fig. 7B). The selective enhancement of b12 neutralization to ASPI-Env, despite increased levels of uncleaved Env, suggests that disrupting the endocytic motif affects only a subset of cleavage-dependent epitopes on the cell surface engaged in cell-to-cell infection.

### Cleavage-deficient Env mediates cell-to-cell transfer of HIV

Cell-to-cell infection is a multistep process initiated by virological synapse (VS) formation, progressing through endocytic uptake into target cells, culminating in Env-mediated fusion, each of which can influence susceptibility to neutralizing antibodies. Because VS formation is the first step and only requires CD4 binding, we asked whether the endocytic motif mutation, which increases the abundance of uncleaved Env on the cell surface, could alter antibody sensitivity at this early step. Importantly, although uncleaved Env is non-fusogenic (58, 59), it can bind CD4 (11, 61), thereby theoretically enabling it to mediate viral particle transfer. To test this, we measured CD4-dependent viral transfer using a short-term assay in which donor cells expressing Gag-fluorescent virions transfer viral particles to target cells (36). This assay specifically measures particle transfer independently of Env-mediated fusion, consistent with previous findings that cell-to-cell infection relies heavily on actin-dependent internalization of viral particles, prior to fusion between the endocytic vesicle and the viral membrane (3, 4, 60, 62, 63).

**Fig 8:**
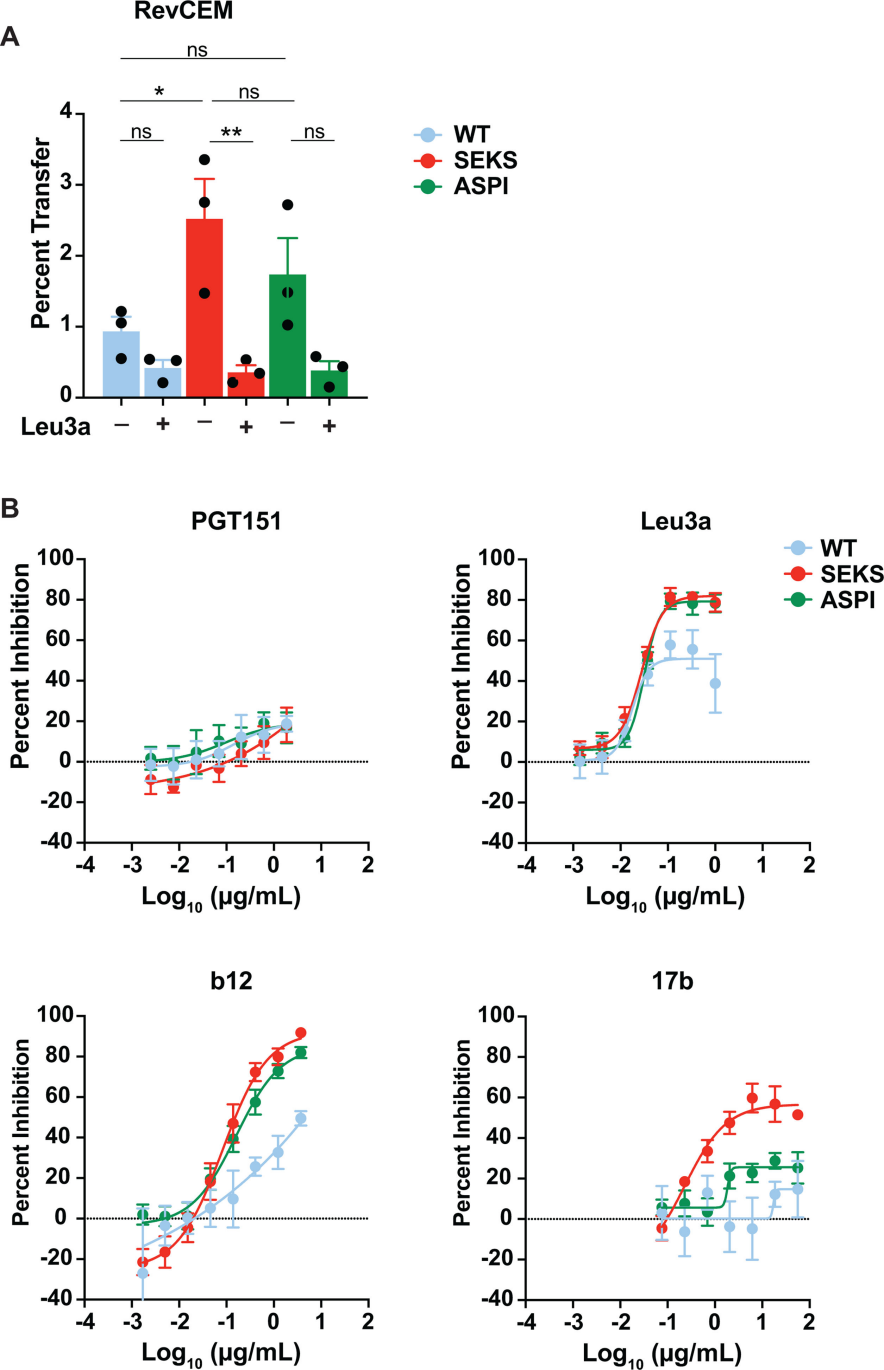
Uncleaved Env participates in CD4-dependent cell-to-cell transfer of HIV-1 to RevCEM. (**A**) Cell-to-cell transfer of WT and mutant GagiCherry_QH0692_ virions from nucleofected Jurkat T cells to RevCEM cells is shown. mCherry expression was used to normalize the nucleofection efficiency in donor cells before co-culturing for 6 h, using flow cytometry. Leu3a at 1 μg/mL was used as a negative control. (**B**) Neutralization of WT and mutant NLCI_QH0692_ cell-to-cell transfer by PGT151, Leu3a, b12, and 17b. The donor cells were incubated with anti-Env mAbs and target cells with Leu3a for 30 min at 37°C before co-culturing cells. All experiments shown represent at least three independent experiments. The mean and SEM data are shown for each curve and bar graph. Statistical significance was performed using Tukey’s multiple comparisons test: *P* < 0.05 = *, *P* < 0.01 = **, *P* < 0.001 = ***, and *P* < 0.0001 = ****, ns = not significant.

To confirm that viral particle transfer is CD4-dependent, target cells were pre-incubated with anti-CD4, Leu3a, which blocks Env interactions at the cell surface. Donor T cells expressing WT-Env or ASPI-Env both showed reproducible transfer of virions to RevCEM target cells (Fig. 8A). Importantly, transfer was reduced in the presence of Leu3a, confirming its CD4 dependence (Fig. 8A). Donor cells expressing SEKS-Env also exhibited CD4-dependent particle transfer, as confirmed by the loss of fluorescent transfer signal observed in the Leu3a-treated condition (Fig. 8A). As SEKS-Env is not fusogenic, viral and target cell membrane fusion is not expected. However, these data show that cleavage-deficient Env can mediate particle transfer, consistent with CD4 engagement playing a key role in VS formation, mediating the transfer of viral particles through endocytosis, a fusion-independent mechanism (3, 4, 60, 62, 63).

To further investigate the antigenic profile of Env engaged in cell-to-cell transfer, we measured neutralization of the transfer assay with the panel of cleavage- and glycan-sensitive antibodies. PGT151 showed no inhibitory activity against transfer with either WT- or mutant-Env donors (Fig. 8B), consistent with its epitope lying outside the CD4 binding site and its inability to block VS formation. In contrast, b12, which targets the CD4 binding site, exhibited enhanced neutralization of SEKS- and ASPI-Env mediated cell-to-cell transfer (Fig. 8B). The increased susceptibility of ASPI-Env relative to WT-Env mirrored the neutralization pattern observed in cell-to-cell infection (Fig. 7B), suggesting that disrupting the endocytic motif alters early CD4-dependent steps. Similarly, 17b and Leu3a also showed greater inhibition of transfer for SEKS- and ASPI-Env than for WT-Env (Fig. 8B). These results further support a role for uncleaved Env in mediating CD4-dependent interactions that initiate VS formation despite its lack of fusogenic capacity.

**Fig 9:**
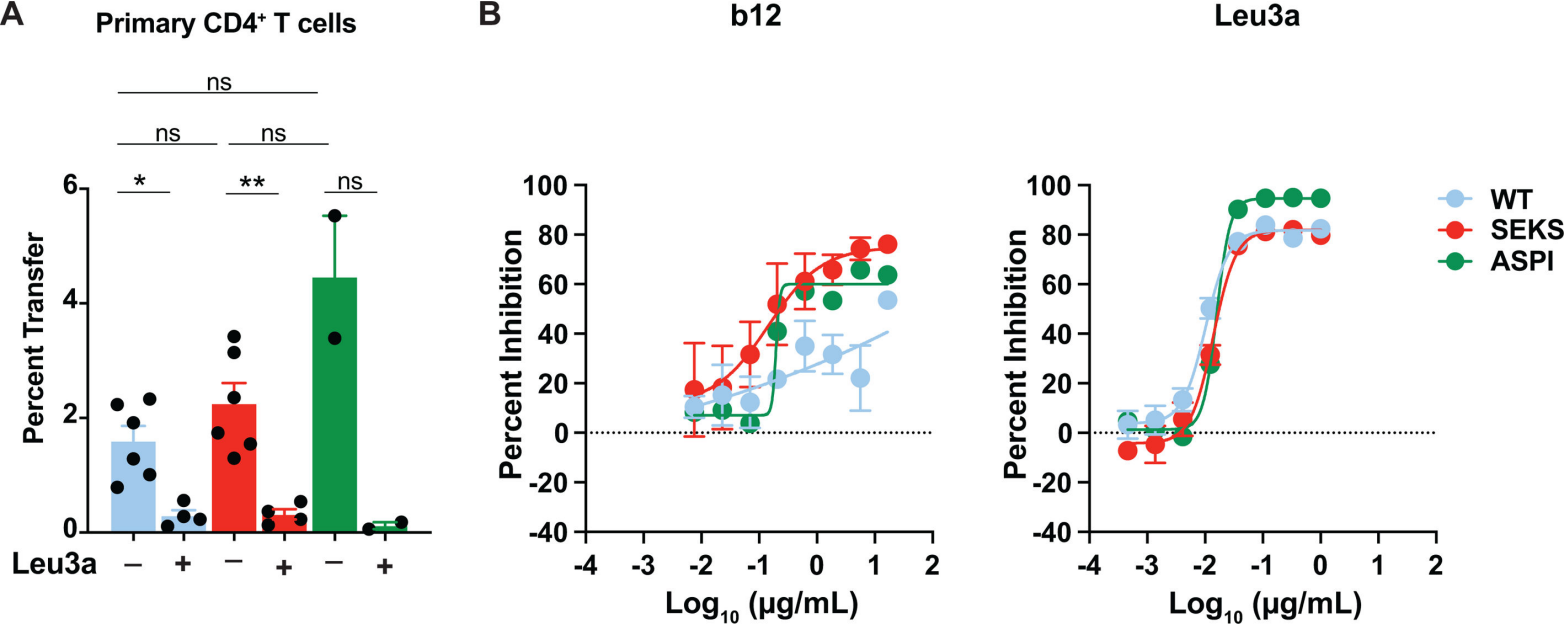
Uncleaved Env facilitates the cell-to-cell transfer of HIV-1 to primary CD4^+^ T cells. (**A**) Cell-to-cell transfer of WT and mutant GagiCherry_QH0692_ virions from nucleofected Jurkat T cells to primary CD4^+^ T cells is shown. mCherry expression was used to normalize the nucleofection efficiency in donor cells before co-culturing for 3 h. Leu3a at 1 μg/mL was used as a negative control. (**B**) Neutralization of WT and mutant NLCI_QH0692_ cell-to-cell transfer by b12 and Leu3a. The donor cells were incubated with anti-Env mAb b12, and the target cells were incubated with anti-CD4 mAb Leu3a for 30 min at 37°C before co-culturing cells. All experiments were performed using target cells from at least two different primary donors, except for the ASPI neutralization experiment, which was conducted with one donor. The mean and SEM data are shown for each curve and bar graph. Statistical significance was performed using Dunnett’s T3 multiple comparisons test: *P* < 0.05 = *, *P* < 0.01 = **, *P* < 0.001 = ***, and *P* < 0.0001 = ****, ns = not significant.

We also performed the cell-to-cell transfer assay with primary CD4^+^ T cells as target cells. As with RevCEM, transfer of fluorescent viral particles was reduced by Leu3a, confirming that the assay specifically measures VS-directed transfer of HIV-1 (Fig. 9A). SEKS-Env facilitated transfer to primary cells comparable to WT-Env, while ASPI-Env exhibited even higher levels (Fig. 9A). Both SEKS- and ASPI-Env also demonstrated increased sensitivity to b12, whereas Leu3a inhibited transfer similarly across all Env variants (Fig. 9B). The enhanced neutralization of SEKS- and ASPI-Env by antibodies with a preference for uncleaved or open Env further suggests that the endocytic motif mutation shifts the composition of cell-surface Env toward uncleaved forms of Env that may participate in the earliest steps of cell-to-cell spread.

## DISCUSSION

HIV-1 vaccine strategies have largely focused on eliciting antibodies against cleaved Env trimers, which predominate the surface of viral particles (24). In contrast, Env displayed on the surface of infected cells is more heterogeneous and includes a larger fraction of uncleaved Env (23, 24). This distinction is important because bNAbs generally exhibit reduced efficacy against cell-to-cell infection compared to cell-free infection (3, 5–8), a difference proposed to arise from the presence of antigenically diverse Env conformations at the plasma membrane and on virions (5, 9). How a selective subset of Env is incorporated into nascent virions remains poorly understood. Given the influence of Env cleavage on antibody recognition and the role of endocytic recycling in Env incorporation onto virus particles, we examined how Env endocytosis affects the balance of cleaved and uncleaved Env at the cell surface, thereby influencing antibody recognition and neutralization during cell-to-cell transmission.

To address this, we disrupted Env’s endocytic motif and assessed surface Env composition. ASPI-Env reduced the proportion of cleaved Env at the cell surface, as shown by both surface immunoprecipitation and ratiometric antibody staining. This effect was not explained solely due to increased total Env expression, as we measured both a decrease in a cleavage-dependent epitope and an increase in epitopes exposed in uncleaved Env. These findings are consistent with a model in which impaired endocytosis promotes the accumulation of uncleaved, more open Env conformations at the cell surface, supporting a role for endocytosis in maintaining a balanced surface population of cleaved and uncleaved trimers.

Despite the observed shift toward more uncleaved Env at the cell surface with ASPI-Env, cell-to-cell neutralization changed little across the selected antibody panel. The notable exception was the CD4-binding site antibody, b12, which showed increased potency against ASPI-Env. While WT-Env exhibited a biphasic inhibition pattern consistent with heterogeneous CD4-binding site accessibility, ASPI-Env yielded a sigmoidal curve, suggesting reduced heterogeneity in CD4 binding site exposure under these conditions.

These findings raised the possibility that uncleaved Env contributes to the early steps of cell-to-cell spread. Although uncleaved Env is non-fusogenic (58, 59), SEKS-Env supported CD4-dependent particle transfer to T cell line and primary T cells, consistent with previous observations that uncleaved Env can bind CD4 (11, 61). These data suggest that both uncleaved and cleaved Env can mediate the initial Env-CD4 interactions required for virological synapse formation and endocytic uptake of viral particles even though cleaved Env is required for membrane fusion. One potential implication of this division of labor is that it may permit particle transfer while limiting donor-target syncytium formation. Consistent with prior work, cleaved Env remains essential for productive infection because membrane fusion is required for viral entry. We hypothesize that uncleaved Env may preferentially persist at the synapse to engage CD4, whereas cleaved Env may be enriched at assembly-associated microdomains (64–66), consistent with reports that Env recycling is not necessary for VS formation, but rapid Env recycling occurs at the synapse (67).

Although a simple model in which endocytic recycling targets cleaved Env to assembly sites could promote distinct roles for cleaved and uncleaved Env in cell-to-cell infection, it does not readily account for the observed increased abundance of uncleaved Env when endocytosis is disrupted. An alternative model is that both cleaved and uncleaved Env undergo endocytosis, with Env conformation influencing intracellular routing. Consistent with the idea that cleavage state may influence Env trafficking, multiple intracellular routes have been described, including retrograde transport to the Golgi, Rab11-dependent recycling to the cell surface, and lysosomal trafficking (64, 68–72). Together, these results support a role for endocytosis in shaping the balance of cleaved and uncleaved Env and underscore the need to define how Env cleavage state and trafficking influence Env presentation during HIV-1 infection.

Diminished Env cleavage also impacted cell-free infection. Although ASPI-Env virions produced in 293T cells remained enriched for cleaved Env, they were more sensitive to neutralization by antibodies in our panel that preferentially recognize uncleaved or open conformations, suggesting that even low-level incorporation of uncleaved Env can measurably alter neutralization despite uncleaved Env not engaging in fusion. One possible explanation is that antibodies bound to uncleaved Env could reduce the clustering of Env trimers induced during CD4 binding, consistent with entry claw models in which multiple Env trimers cooperate during entry (73–76). Additionally, because 293T cells are classified as permissive to mutations in Env’s cytoplasmic tail that disrupt incorporation into virus particles, this system could underestimate effects observed in more physiologic producer cells.

This study has limitations, including our focus on a single transmitted/founder Env clone and use of an antibody panel enriched for cleavage- and glycan-sensitive epitopes. Our antibody selection was intentional, because these reagents have well-defined preferences for cleaved vs uncleaved Env, enabling a focused assessment of cleavage-associated antigenicity. Because the endocytic motif examined is highly conserved across HIV-1 lineages (77), these findings may be broadly relevant; however, additional studies will be needed to determine how generalizable these effects are across diverse transmitted/founder Envs and producer cell types.

Additionally, when assessing the CD4-dependence of cell-to-cell transfer assays, we noted incomplete neutralization by Leu3a, particularly when RevCEM cells were used as targets with WT-Env. We speculate that the 6-h coculture experiment, Leu3a may decrease surface CD4 levels by increasing internalization (78, 79), thereby additionally limiting HIV internalization after cell-cell engagement. It may follow that the ASPI-Env, which has a higher density of surface Env, is less sensitive to lower levels of CD4 on the target cell. This pattern was also apparent but less prominent in primary CD4^+^ T cells which express higher levels of surface CD4 (80). Additional studies will be required to determine how CD4 abundance influences the neutralization efficiency of cell-to-cell infection.

In conclusion, our results support a role for Env endocytosis in shaping cleavage and glycosylation-associated antigenicity during both cell-free and cell-to-cell transmission, with consequences for neutralizing antibody sensitivity. These findings have implications for vaccine designs, because strategies focused exclusively on cleaved Env may not fully capture antigenic states present on infected cells, including uncleaved Env species that can participate in the early steps of cell-to-cell spread (81–86). Because antibody engagement of infected cells also contributes to effector functions such as antibody dependent cellular cytotoxicity (ADCC), antibody dependent cellular phagocytosis (ADCP), or complement activation, it will be important to consider how Env trafficking pathways shape the range of Env conformations displayed at the cell surface. Future vaccines and therapeutics may benefit from targeting a broader range of Env conformations and accounting for endocytic recycling pathways that shape Env antigenicity at the cell surface.

## Data Availability

Supporting data for the study are available online through the Figshare repository (DOI: 10.6084/m9.figshare.30251974). Uncropped immunoblot scans are provided as Source Data. Data files containing primary data for densitometry, cell-free, and cell-to-cell neutralization assays, as well as the R code script used for ratiometric fluorescence analysis, are archived on Figshare. No large-scale sequencing or structural data sets were generated. Materials (cell lines, plasmids, and viral constructs) are available from the corresponding author under a standard MTA.
